# Supplementary material for: Modeling glioblastoma heterogeneity as a dynamic network of cell states
Source: Mol Syst Biol. 2021 Sep 16;17(9):e10105. doi: 10.15252/msb.202010105 (PMC8444284; doi:10.15252/msb.202010105)
Supplement: Supplementary file 5 — Source Data for Figure 3 [file MSB-17-e10105-s001.zip › Figure3A_sourcedata/GSEA_3065/nosigdb_state4.GseaPreranked.1622718430994/FELDMAN_2020_S.html]

Details for gene set FELDMAN\_2020\_S[GSEA]

|  || Dataset | state4 |
| Phenotype | NoPhenotypeAvailable |
| Upregulated in class | na\_neg |
| GeneSet | FELDMAN\_2020\_S |
| Enrichment Score (ES) | -0.7476101 |
| Normalized Enrichment Score (NES) | -2.8187804 |
| Nominal p-value | 0.0 |
| FDR q-value | 0.0 |
| FWER p-Value | 0.0 |
Table: GSEA Results Summary

  

Fig 1: Enrichment plot: FELDMAN\_2020\_S      
 Profile of the Running ES Score & Positions of GeneSet Members on the Rank Ordered List

  

| PROBE | GENE SYMBOL | GENE\_TITLE | RANK IN GENE LIST | RANK METRIC SCORE | RUNNING ES | CORE ENRICHMENT || 1 | SAT1 |  |  | 4 | 0.669 | 0.0169 | No |
| 2 | EEF1A1 |  |  | 12 | 0.471 | 0.0281 | No |
| 3 | FTL |  |  | 13 | 0.466 | 0.0404 | No |
| 4 | SOX4 |  |  | 18 | 0.402 | 0.0503 | No |
| 5 | MARCKS |  |  | 27 | 0.363 | 0.0585 | No |
| 6 | SESN3 |  |  | 68 | 0.278 | 0.0593 | No |
| 7 | RPS27L |  |  | 149 | 0.223 | 0.0521 | No |
| 8 | BEX4 |  |  | 220 | 0.194 | 0.0457 | No |
| 9 | LYPD1 |  |  | 264 | 0.183 | 0.0435 | No |
| 10 | PAIP2 |  |  | 336 | 0.168 | 0.0363 | No |
| 11 | TSC22D1 |  |  | 369 | 0.160 | 0.0353 | No |
| 12 | PHLDA1 |  |  | 464 | 0.142 | 0.0236 | No |
| 13 | DDIT4 |  |  | 493 | 0.138 | 0.0227 | No |
| 14 | EEF2 |  |  | 729 | 0.110 | -0.0128 | No |
| 15 | YPEL5 |  |  | 783 | 0.105 | -0.0187 | No |
| 16 | ZFAND6 |  |  | 978 | 0.090 | -0.0480 | No |
| 17 | HES4 |  |  | 1197 | 0.076 | -0.0817 | No |
| 18 | RAB13 |  |  | 1270 | 0.072 | -0.0915 | No |
| 19 | TGIF1 |  |  | 1699 | 0.055 | -0.1600 | No |
| 20 | CALD1 |  |  | 2682 | 0.031 | -0.3197 | No |
| 21 | BAMBI |  |  | 3437 | 0.014 | -0.4426 | No |
| 22 | BTG1 |  |  | 4064 | -0.013 | -0.5445 | No |
| 23 | ARL6IP5 |  |  | 4364 | -0.033 | -0.5925 | No |
| 24 | TFDP1 |  |  | 4389 | -0.035 | -0.5955 | No |
| 25 | YWHAH |  |  | 4595 | -0.051 | -0.6277 | No |
| 26 | RMI2 |  |  | 4623 | -0.053 | -0.6307 | No |
| 27 | EIF1AX |  |  | 4700 | -0.060 | -0.6416 | No |
| 28 | MEST |  |  | 4739 | -0.063 | -0.6461 | No |
| 29 | LIMS1 |  |  | 4775 | -0.066 | -0.6501 | No |
| 30 | HNRNPF |  |  | 4783 | -0.066 | -0.6495 | No |
| 31 | POLR3K |  |  | 4904 | -0.075 | -0.6671 | No |
| 32 | SCRG1 |  |  | 4970 | -0.080 | -0.6757 | No |
| 33 | HSPA8 |  |  | 5041 | -0.086 | -0.6848 | No |
| 34 | GGCT |  |  | 5073 | -0.089 | -0.6876 | No |
| 35 | CARHSP1 |  |  | 5107 | -0.092 | -0.6905 | No |
| 36 | TPM1 |  |  | 5108 | -0.092 | -0.6881 | No |
| 37 | UNG |  |  | 5148 | -0.096 | -0.6920 | No |
| 38 | TAGLN |  |  | 5209 | -0.102 | -0.6991 | No |
| 39 | RNASEH2A |  |  | 5241 | -0.105 | -0.7014 | No |
| 40 | PPIF |  |  | 5337 | -0.112 | -0.7140 | No |
| 41 | RPA2 |  |  | 5372 | -0.115 | -0.7165 | No |
| 42 | EEF1E1 |  |  | 5432 | -0.122 | -0.7229 | No |
| 43 | RFC3 |  |  | 5450 | -0.124 | -0.7224 | No |
| 44 | DCTPP1 |  |  | 5588 | -0.143 | -0.7411 | No |
| 45 | TEX30 |  |  | 5629 | -0.148 | -0.7437 | Yes |
| 46 | EXOSC9 |  |  | 5643 | -0.151 | -0.7419 | Yes |
| 47 | MCM6 |  |  | 5644 | -0.151 | -0.7379 | Yes |
| 48 | CCDC34 |  |  | 5647 | -0.152 | -0.7342 | Yes |
| 49 | HSPB11 |  |  | 5707 | -0.161 | -0.7396 | Yes |
| 50 | MSH2 |  |  | 5711 | -0.162 | -0.7359 | Yes |
| 51 | CACYBP |  |  | 5713 | -0.162 | -0.7318 | Yes |
| 52 | MAGOHB |  |  | 5718 | -0.164 | -0.7281 | Yes |
| 53 | CDCA7L |  |  | 5720 | -0.164 | -0.7240 | Yes |
| 54 | EIF4EBP1 |  |  | 5747 | -0.171 | -0.7237 | Yes |
| 55 | SLBP |  |  | 5748 | -0.172 | -0.7192 | Yes |
| 56 | EXOSC8 |  |  | 5751 | -0.173 | -0.7150 | Yes |
| 57 | MALAT1 |  |  | 5783 | -0.179 | -0.7154 | Yes |
| 58 | NOVA1 |  |  | 5789 | -0.181 | -0.7114 | Yes |
| 59 | SIVA1 |  |  | 5791 | -0.181 | -0.7068 | Yes |
| 60 | MSH6 |  |  | 5799 | -0.182 | -0.7032 | Yes |
| 61 | HAT1 |  |  | 5803 | -0.184 | -0.6989 | Yes |
| 62 | MIS18A |  |  | 5816 | -0.187 | -0.6959 | Yes |
| 63 | CCNE2 |  |  | 5827 | -0.190 | -0.6926 | Yes |
| 64 | PA2G4 |  |  | 5841 | -0.193 | -0.6896 | Yes |
| 65 | PTMA |  |  | 5852 | -0.195 | -0.6861 | Yes |
| 66 | CDCA4 |  |  | 5861 | -0.197 | -0.6823 | Yes |
| 67 | HNRNPAB |  |  | 5862 | -0.197 | -0.6771 | Yes |
| 68 | CDCA7 |  |  | 5863 | -0.198 | -0.6719 | Yes |
| 69 | ANP32B |  |  | 5890 | -0.204 | -0.6708 | Yes |
| 70 | C19orf48 |  |  | 5894 | -0.205 | -0.6659 | Yes |
| 71 | HAUS8 |  |  | 5897 | -0.206 | -0.6608 | Yes |
| 72 | SNRPB |  |  | 5908 | -0.209 | -0.6570 | Yes |
| 73 | BRCA2 |  |  | 5910 | -0.210 | -0.6516 | Yes |
| 74 | SNRPD1 |  |  | 5911 | -0.210 | -0.6461 | Yes |
| 75 | FAM111B |  |  | 5918 | -0.211 | -0.6416 | Yes |
| 76 | ACAT2 |  |  | 5931 | -0.215 | -0.6379 | Yes |
| 77 | RANBP1 |  |  | 5939 | -0.218 | -0.6333 | Yes |
| 78 | PSMC3IP |  |  | 5951 | -0.222 | -0.6293 | Yes |
| 79 | POLD3 |  |  | 5957 | -0.225 | -0.6242 | Yes |
| 80 | CENPA |  |  | 5960 | -0.226 | -0.6186 | Yes |
| 81 | PGP |  |  | 5966 | -0.227 | -0.6134 | Yes |
| 82 | NCL |  |  | 5974 | -0.228 | -0.6086 | Yes |
| 83 | CENPV |  |  | 5984 | -0.232 | -0.6040 | Yes |
| 84 | USP1 |  |  | 5986 | -0.233 | -0.5980 | Yes |
| 85 | AKAP12 |  |  | 5988 | -0.234 | -0.5920 | Yes |
| 86 | RAN |  |  | 5990 | -0.235 | -0.5860 | Yes |
| 87 | RPA3 |  |  | 6014 | -0.244 | -0.5834 | Yes |
| 88 | AURKA |  |  | 6026 | -0.248 | -0.5786 | Yes |
| 89 | CTNNAL1 |  |  | 6027 | -0.249 | -0.5721 | Yes |
| 90 | VRK1 |  |  | 6034 | -0.256 | -0.5664 | Yes |
| 91 | FEN1 |  |  | 6035 | -0.256 | -0.5596 | Yes |
| 92 | DNAJC9 |  |  | 6037 | -0.260 | -0.5530 | Yes |
| 93 | SMC3 |  |  | 6041 | -0.265 | -0.5465 | Yes |
| 94 | CDC45 |  |  | 6063 | -0.276 | -0.5427 | Yes |
| 95 | MCM5 |  |  | 6069 | -0.278 | -0.5362 | Yes |
| 96 | GMNN |  |  | 6080 | -0.285 | -0.5303 | Yes |
| 97 | FBXO5 |  |  | 6084 | -0.287 | -0.5233 | Yes |
| 98 | DEK |  |  | 6092 | -0.292 | -0.5167 | Yes |
| 99 | RFC2 |  |  | 6095 | -0.294 | -0.5094 | Yes |
| 100 | CDC6 |  |  | 6103 | -0.299 | -0.5026 | Yes |
| 101 | DUT |  |  | 6107 | -0.303 | -0.4952 | Yes |
| 102 | MCM10 |  |  | 6110 | -0.305 | -0.4875 | Yes |
| 103 | SLC20A1 |  |  | 6112 | -0.306 | -0.4796 | Yes |
| 104 | MCM3 |  |  | 6113 | -0.307 | -0.4716 | Yes |
| 105 | ASF1B |  |  | 6127 | -0.320 | -0.4653 | Yes |
| 106 | SRSF2 |  |  | 6128 | -0.321 | -0.4569 | Yes |
| 107 | TMEM97 |  |  | 6131 | -0.324 | -0.4487 | Yes |
| 108 | LMNB1 |  |  | 6134 | -0.325 | -0.4405 | Yes |
| 109 | CCNB2 |  |  | 6140 | -0.329 | -0.4327 | Yes |
| 110 | TK1 |  |  | 6144 | -0.334 | -0.4244 | Yes |
| 111 | ARL6IP1 |  |  | 6145 | -0.336 | -0.4156 | Yes |
| 112 | SRSF7 |  |  | 6150 | -0.343 | -0.4072 | Yes |
| 113 | DLGAP5 |  |  | 6156 | -0.345 | -0.3990 | Yes |
| 114 | ATAD5 |  |  | 6161 | -0.350 | -0.3904 | Yes |
| 115 | CDCA3 |  |  | 6165 | -0.355 | -0.3816 | Yes |
| 116 | CENPK |  |  | 6169 | -0.363 | -0.3725 | Yes |
| 117 | MCM4 |  |  | 6171 | -0.372 | -0.3629 | Yes |
| 118 | PCNA |  |  | 6173 | -0.374 | -0.3533 | Yes |
| 119 | CENPM |  |  | 6179 | -0.386 | -0.3440 | Yes |
| 120 | DNMT1 |  |  | 6182 | -0.396 | -0.3339 | Yes |
| 121 | CHAF1A |  |  | 6183 | -0.396 | -0.3235 | Yes |
| 122 | RFC4 |  |  | 6185 | -0.399 | -0.3132 | Yes |
| 123 | MANF |  |  | 6186 | -0.400 | -0.3027 | Yes |
| 124 | NASP |  |  | 6187 | -0.403 | -0.2921 | Yes |
| 125 | GINS2 |  |  | 6194 | -0.420 | -0.2820 | Yes |
| 126 | ZWINT |  |  | 6195 | -0.425 | -0.2709 | Yes |
| 127 | BRCA1 |  |  | 6197 | -0.435 | -0.2596 | Yes |
| 128 | RRM2 |  |  | 6199 | -0.438 | -0.2483 | Yes |
| 129 | DHFR |  |  | 6200 | -0.441 | -0.2367 | Yes |
| 130 | CCND1 |  |  | 6203 | -0.454 | -0.2251 | Yes |
| 131 | ESCO2 |  |  | 6206 | -0.463 | -0.2133 | Yes |
| 132 | CLSPN |  |  | 6218 | -0.490 | -0.2022 | Yes |
| 133 | HELLS |  |  | 6221 | -0.501 | -0.1894 | Yes |
| 134 | TYMS |  |  | 6222 | -0.501 | -0.1762 | Yes |
| 135 | SMC2 |  |  | 6229 | -0.518 | -0.1636 | Yes |
| 136 | ORC6 |  |  | 6238 | -0.585 | -0.1495 | Yes |
| 137 | UBE2T |  |  | 6240 | -0.593 | -0.1341 | Yes |
| 138 | ATAD2 |  |  | 6243 | -0.607 | -0.1185 | Yes |
| 139 | PTTG1 |  |  | 6245 | -0.622 | -0.1023 | Yes |
| 140 | CDK1 |  |  | 6246 | -0.627 | -0.0858 | Yes |
| 141 | MAD2L1 |  |  | 6247 | -0.628 | -0.0693 | Yes |
| 142 | CENPU |  |  | 6248 | -0.633 | -0.0527 | Yes |
| 143 | H2AFZ |  |  | 6250 | -0.651 | -0.0358 | Yes |
| 144 | MCM7 |  |  | 6252 | -0.665 | -0.0185 | Yes |
| 145 | HMGB2 |  |  | 6258 | -0.766 | 0.0008 | Yes |
Table: GSEA details [plain text format]

  

Fig 2: FELDMAN\_2020\_S: Random ES distribution      
 Gene set null distribution of ES for **FELDMAN\_2020\_S**
